# Comparison of Pharmacokinetics and Anti-Pulmonary Fibrosis-Related Effects of Sulforaphane and Sulforaphane *N*-acetylcysteine

**DOI:** 10.3390/pharmaceutics13070958

**Published:** 2021-06-25

**Authors:** Eun Suk Son, Xiang Fei, Jin-Ha Yoon, Seung-Yong Seo, Han-Joo Maeng, Sung Hwan Jeong, Yu Chul Kim

**Affiliations:** 1 Department of Medicine, Gachon University Gil Medical Center, Incheon 21565, Korea; eunsuk0607@hanmail.net; 2 Department of Pharmacy, College of Pharmacy, Gachon University, Incheon 21936, Korea; mrfeixiang@gmail.com (X.F.); jinha89@daum.net (J.-H.Y.); syseo@gachon.ac.kr (S.-Y.S.); hjmaeng@gachon.ac.kr (H.-J.M.); 3 Department of Pharmaceutical Engineering, Inje University, Gimhae 50834, Korea; 4 Institute of Digital Anti-Aging Healthcare, Inje University, Gimhae 50834, Korea

**Keywords:** sulforaphane, sulforaphane *N*-acetylcysteine, anti-pulmonary fibrosis effect, pharmacokinetics, metabolic stability

## Abstract

Sulforaphane (SFN), belonging to the isothiocyanate family, has received attention owing to its beneficial activities, including chemopreventive and antifibrotic effects. As sulforaphane *N*-acetylcysteine (SFN-NAC), a major sulforaphane metabolite, has presented similar pharmacological activities to those of SFN, it is crucial to simultaneously analyze the pharmacokinetics and activities of SFN and SFN-NAC, to comprehensively elucidate the efficacy of SFN-containing products. Accordingly, the anti-pulmonary fibrotic effects of SFN and SFN-NAC were assessed, with simultaneous evaluation of permeability, metabolic stability, and in vivo pharmacokinetics. Both SFN and SFN-NAC decreased the levels of transforming growth factor-β1-induced fibronectin, alpha-smooth muscle actin, and collagen, which are major mediators of fibrosis, in MRC-5 fibroblast cells. Regarding pharmacokinetics, SFN and SFN-NAC were metabolically unstable, especially in the plasma. SFN-NAC degraded considerably faster than SFN in plasma, with SFN being formed from SFN-NAC. In rats, SFN and SFN-NAC showed a similar clearance when administered intravenously; however, SFN showed markedly superior absorption when administered orally. Although the plasma SFN-NAC concentration was low owing to poor absorption following oral administration, SFN-NAC was converted to SFN in vivo, as in plasma. Collectively, these data suggest that SFN-NAC could benefit a prodrug formulation strategy, possibly avoiding the gastrointestinal side effects of SFN, and with improved SFN-NAC absorption.

## 1. Introduction

Pulmonary fibrosis (PF) is pathologically characterized by accumulation of fibroblastic foci, excessive matrix deposition, and aberrant remodeling that result in impaired gas exchange, diminished lung capacity, and eventually death [1,2]. Fibroblasts, the major mesenchymal cells in the lung, are activated by fibrogenic growth factors such as tissue growth factor and transforming growth factor (TGF)-β1, which promotes differentiation into myofibroblasts. Furthermore, myofibroblast activation induces excessive accumulation of ECM components, which destroy the alveolar structure [3,4].

Isothiocyanates are sulfur-containing phytochemicals with the general formula R-N=C=S. Sulforaphane (1-isothiocyanato-4-methylsulfinylbutane; SFN), known to belong to this group of chemicals, is mainly present in broccoli as a glucosinolate conjugate. On the crushing and chewing of vegetables, the glucosinolate precursor of SFN is metabolized to SFN via enzymatic hydrolysis by myrosinase [5]. Previously, several studies have shown that SFN mediates chemopreventive effects by blocking the initiation of carcinogenesis, mediated by inhibiting enzymes that convert procarcinogens to carcinogens and inducing phase II enzymes that conjugate carcinogens and facilitate their excretion from the body [6,7]. Furthermore, SFN has shown direct anticancer effects by inhibiting proliferation and inducing apoptosis in tumor cell lines and animal models [8,9,10,11]. More recently, it was observed that SFN treatment attenuated the transforming growth factor (TGF)-beta1-induced expression of fibrosis-related proteins, including fibronectin (FN), type I collagen (COL1A1), type IV collagen (COL4A1), and alpha-smooth muscle actin (α-SMA) in the lung-derived cells A549 and MRC-5, in vitro [12]. Moreover, bleomycin-induced fibrosis was reportedly reduced in a mouse model following SFN treatment [12]. Following absorption, SFN is conjugated with glutathione and further metabolized to sulforaphane-cysteine, sulforaphane-cysteine-glycine, and sulforaphane *N*-acetylcysteine (SFN-NAC) [13,14]. SFN conjugates are converted to their parent form to attain an equilibrium state under physiological conditions and could be considered prodrugs of SFN. Among known SFN conjugates, SFN-NAC is the most stable conjugate and the primary form excreted in urine [15,16]. Furthermore, it has been reported that SFN-NAC has chemopreventive and anticancer activities, although SFN-NAC is a phase II enzyme-mediated metabolite [17,18,19]. Therefore, the pharmacokinetics of both SFN and SFN-NAC should be simultaneously investigated to clarify the pharmacological effects of SFN in terms of its antifibrotic effects.

Several studies have reported the pharmacokinetics of SFN [20,21,22]. SFN is well absorbed in the intestine, with an absolute bioavailability of approximately 82% at 0.5 mg/kg; however, the bioavailability decreases as the dose increases to 5 mg/kg, suggesting non-linear pharmacokinetics in rats [21]. However, the pharmacokinetics of SFN are highly variable and inconsistent in previous reports. For example, the plasma concentration profiles of SFN are highly variable between studies, and the half-life of SFN ranged between 7.6 and 65.6 h after intravenous administration in rats [21,22]. Moreover, to the best of our knowledge, the pharmacokinetics of SFN-NAC has not yet been independently evaluated. The non-linear pharmacokinetics and high variability in pharmacokinetic parameters of SFN could induce inter-subject variability for the pharmacological effects of SFN and SFN-NAC. In the present study, we compared the in vitro anti-pulmonary fibrosis effects of SFN and SFN-NAC using human lung fibroblast cells (MRC-5). In addition, the pharmacokinetics of SFN in the micro-dose range, which seemed to follow linear kinetics, was investigated. In addition, the pharmacokinetics of SFN-NAC, an active metabolite of SFN, was evaluated along with the formed SFN.

## 2. Materials and Methods

### 2.1. Chemicals and Reagents

**Figure 1:**
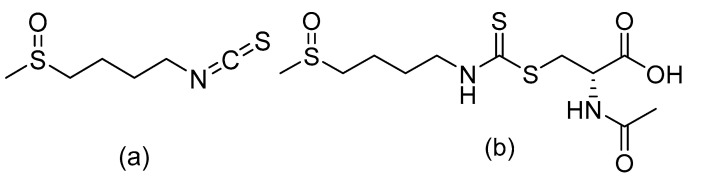
Chemical structures of (**a**) Sulforaphane (SFN) and (**b**) Sulforaphane *N*-acetylcysteine (SFN-NAC).

The structures of SFN and SFN-NAC are shown in Figure 1. SFN (cat. # S699115) and SFN-NAC (cat. # S699120) were purchased from TRC (North York, ON, Canada). Human and rat plasma were obtained from XenoTech (Kansas City, KS, USA). Human and rat liver microsomes were purchased from BD Biosciences (Corning, NY, USA), and water was purified using the aquaMAX™ ultra-pure water purification system (YL Instruments, Anyang, Korea). All other chemicals and solvents were of reagent or HPLC grade and were used without further purification.

### 2.2. In Vitro Anti-Lung Fibrosis Effects

#### 2.2.1. Cell Viability Assay

Human lung fibroblast cells (MRC-5) were purchased from the Korean Cell Line Bank and cultured in Dulbecco’s modified Eagle’s medium (WelGene, Gyeongsan, Korea) supplemented with 100 μg/mL streptomycin (WelGene), 100 U/mL penicillin (WelGene), and 10% fetal bovine serum (WelGene). Then, cells were treated with different concentrations of SFN (10 and 20 μM) and SFN-NAC (10 and 20 μM) for the indicated time periods. The cells were also treated with 0.1% dimethyl sulfoxide (DMSO) as a vehicle control.

In brief, cells were seeded into 96-well plates and exposed to various concentrations of SFN and SFN-NAC for 24–72 h prior to the addition of 10 μL MTT solution (3-(4,5-dimethylthiazol-2-yl)-2,5-diphenyltetrazolium bromide; 5 mg/mL in DPBS) to each well. After 4 h of incubation at 37 °C, the medium was gently removed, and 100 μL DMSO was added. The absorbance was measured at 550 nm using a microplate reader (Thermo LabSystems, Helsinki, Finland).

#### 2.2.2. Quantitative Reverse Transcription-PCR (qRT-PCR) Analysis

Total RNA was extracted using RNAiso Plus reagent (Takara Bio, Shiga, Japan), and cDNA was synthesized from total RNA using a Prime Script RT reagent kit (Takara Bio). qRT-PCR reactions were performed using a SYBR Premix kit (Takara Bio) and run on a CFX96 detection system (BioRad, Hercules, CA, USA). GAPDH was used as an internal control, and all experiments were performed in triplicate. The expression levels were calculated from the PCR profiles of each sample using the threshold cycle (Ct), corresponding to the cycle with a statistically significant increase in fluorescence. The Ct values for the endogenous control (GAPDH) were subtracted from those of the corresponding sample to correct for differences in the amount of total cDNA in the starting reaction.

The primers used for qRT-PCR were as follows: 5′-CCATCGCAAACCGCTGCCAT-3′ (FN-F) and 5′-AACACTTCTCAGCTATGGGCTT-3′ (FN-R); 5′-ACTGAGCGTGGCTATTCCTCCGTT-3′ (α-SMA-F) and 5′-GCAGTGGCCATCTCATTTTCA-3′ (α-SMA-R); 5′-TGGCCAAGAAGACATCCCTGAAGT-3′ (COL1A1-F) and 5′-ACATCAGGTTTCCACGTCTCACCA-3′ (COL1A1-R); 5′-TGTGGGCCAGCCAGGCATTG-3′ (COL4A1-F) and 5′-CAGGGGGTCCGATCGCTCCA-3′ (COL4A1-R); 5′-TTGGTATCGTGGAAGGACTCA-3′ (GAPDH-F) 5′-TGTCATCATATTTGGCAGGTTT-3′ (GAPDH-R).

#### 2.2.3. Western Blot Analysis

In brief, cells were lysed in radioimmunoprecipitation assay (RIPA) buffer (150 mM NaCl, 50 mM Tris-HCl at pH 8.0, 0.1% SDS, 0.5% deoxycholate, 1% NP-40) with 1X protease inhibitors. Protein concentration was determined using a Pierce BCA protein assay (Thermo Fisher Scientific, Rockford, IL, USA). Then, protein was separated by 8–10% sodium dodecyl sulfate-polyacrylamide gel electrophoresis and transferred to a PVDF membrane (Millipore, Bedford, MA, USA). After blocking with 5% non-fat milk, the membrane was incubated with primary antibodies. The primary antibodies used were anti-fibronectin (Santa Cruz Biotechnology, Santa Cruz, CA, USA, sc-8422, 1:1000), anti-type 1 collagen (COL1A1) (Abcam, Cambridge, UK, ab90395, 1:1000), anti-type 4 collagen (COL4A1) (Abcam, ab135802, 1:1000), anti-alpha-SMA (Abcam, ab5694, 1:1000), and anti-GAPDH (Abfrontier, Seoul, Korea, LF-PA0018, 1:2000). The membrane was washed and then incubated with horseradish peroxidase (HRP)-conjugated anti-mouse (Genetex, Hsinchu, Taiwan, GTX213110-01) or anti-rabbit secondary antibodies (Genetex, GTX213111-01). Antibody-bound protein was detected using a Western BLoT Hyper HRP Substrate kit (Takara Bio, Shiga, Japan). Band intensities were normalized to those of GAPDH using the ImageJ software (National Institutes of Health, Bethesda, MD, USA). All experiments were independently performed four times.

### 2.3. In Vitro Pharmacokinetic Study

#### 2.3.1. Plasma Stability Assay

The stability of SFN and SFN-NAC was investigated in human and rat plasma. Test compounds (1 μM) were incubated with rat and human plasma. The amount remaining (%) was determined at 0, 15, 30, 60, 120, 180, and 240 min using liquid chromatography-tandem mass spectrometry (LC-MS/MS).

#### 2.3.2. Liver Microsomal Metabolic Stability

The metabolic stability of SFN and SFN-NAC was evaluated in human and rat liver microsomes as previously reported [23], with minor modifications. Briefly, test compounds (1 μM) were incubated with rat and human liver microsomes (final concentration 1 mg/mL protein) containing 1 mM NADPH. The amount remaining was determined at 0, 15, 30, 60, 120, 180, and 240 min using LC-MS/MS.

#### 2.3.3. Apparent Permeability for Direction of Absorption in Caco-2 Cells

Apparent permeability in the direction of absorption (apical-to-basolateral side) was determined as previously reported [24]. Briefly, test compounds (54 μM) were loaded on the apical side of the Caco-2 cell monolayer, which was cultured in a transwell plate for 21 days after seeding cells at a density of 8 × 10^4^ cells/cm^2^. Next, to measure the absorptive transport of drugs, the inserts were moved to a well containing fresh transport media, replaced every 30 min for 2 h. The cumulative amount on the basolateral side was calculated following sample analysis using LC-MS/MS. Finally, the apparent permeability of the apical-to-basolateral side was calculated by dQ/dt, the rate of appearance of the drug on the receiver side, divided by A × 60 × Co, where A is the surface area of the Caco-2 cell monolayer (1.12 cm^2^) and Co is the loading concentration of the drug on the apical side.

### 2.4. In Vivo Pharmacokinetic Study at a Micro-Dose Range

#### 2.4.1. Animal Study

For SFN and SFN-NAC, pharmacokinetics was assessed in male Sprague-Dawley rats (7–8 weeks old, 220–280 g) obtained from Orient Bio Inc. (Seongnam, Korea). Rats were maintained under a standard 12:12 h light/dark cycle for 1 week. All experiments complied with the guidelines for animal care and use issued by Gachon University, and experimental animal protocols were reviewed and approved by the Animal Care and Use Committee of Gachon University (#GIACUC-R2018004, approval date: 11 May 2018).

For the intravenous study, overnight fasted rats were intramuscularly anesthetized using tiletamine/zolazepam (Zoletil; Vibrac Laboratories, Carros, France) (20 mg/kg). Then, the femoral vein and artery were surgically cannulated with polyethylene tubing (PE50; Clay Adams, Parsippany, NJ, USA) for intravenous administration of test compounds and blood sampling, respectively. The cannula was flushed with 20 IU/mL heparinized saline to prevent clotting. After the rats had recovered from anesthesia, SFN or SFN-NAC was injected intravenously at 0.1 mg/kg (*n* = 4). Blood samples (100 μL) were collected from the femoral artery 1, 5, 15, 30, 45, 60, 120, 180, and 240 min after administration, then immediately centrifuged at 14,000 rpm for 15 min at 4 °C, and the plasma obtained was stored at −70 °C prior to LC-MS/MS analysis. For the oral study, the femoral vein was cannulated for blood sampling, followed by administration of SFN (0.1, 0.2, and 0.5 mg/kg) or SFN-NAC (0.5 mg/kg) using oral gavage. Blood samples (100 μL) were collected from the femoral artery 15, 30, 45, 60, 120, 180, 240, 480, and 1440 min after administration and processed in the same way as in the intravenous study.

#### 2.4.2. Pharmacokinetic Analysis

Noncompartmental analysis of SFN and SFN-NAC was performed using the WinNonlin program (Ver. 5.0.1., Pharsight Corporation, Mountain View, CA, USA) to calculate the following pharmacokinetic parameters: area under the plasma concentration-time curve from time zero to infinity (AUC_inf_) or to the last measured time (AUC_last_), total body clearance (CL), elimination half-life (t_1/2_), volume of distribution at steady-state (V_ss_), and mean residence time (MRT). The AUC was calculated using the trapezoidal rule extrapolation method. The maximum plasma concentration (C_max_) and time to reach C_max_ (T_max_) were directly determined from the experimental data, and the area from the last datum point to time infinity (for the calculation of AUC_inf_) was estimated by dividing the last measured plasma concentration by the terminal-phase rate constant. The absolute bioavailability (F%) was calculated as the dose-normalized ratio of the AUC after oral and intravenous administration.

### 2.5. LC-MS/MS Analysis

In the present study, sample concentrations from in vitro or in vivo pharmacokinetic studies were determined using LC-MS/MS in positive electrospray ionization mode. The LC-MS/MS system consisted of an Agilent 6490 QQQ with a 1290 Infinity HPLC system (Agilent Technologies, Santa Clara, CA, USA). To separate the analytes, SFN and SFN-NAC, from endogenous substances, a SynergiTM 4 μm polar RP 80A column (150 × 2.0 mm, Phenomenex, Torrance, CA, USA) was employed. The LC conditions were as follows: mobile phase, 0.1% formic acid and acetonitrile (50:50); flow rate, 0.2 mL/min; injection volume, 2 μL. Multiple reaction monitoring was achieved with *m*/*z* 178.1→114.1 for SFN, *m*/*z* 340.9→178.1 for SFN-NAC, and *m*/*z* 176.1→111.9 for an internal standard (sulforaphene). Data acquisition and processing were performed using the MassHunter software (version A.06.00, Agilent Technologies). The limit of quantitation was 0.2 ng/mL for SFN and 0.5 ng/mL for SFN-NAC.

### 2.6. Statistical Analyses

Data are expressed as mean ± standard deviation (SD) of at least three independent experiments. For statistical analysis, one-way analysis of variance (ANOVA) with post-hoc Dunnett’s test was performed using GraphPad Prism software (San Diego, CA, USA). *p*-values less than 0.05 were considered statistically significant.

## 3. Results

### 3.1. In Vitro Anti-Fibrosis Effects of SFN and SFN-NAC

**Figure 2:**
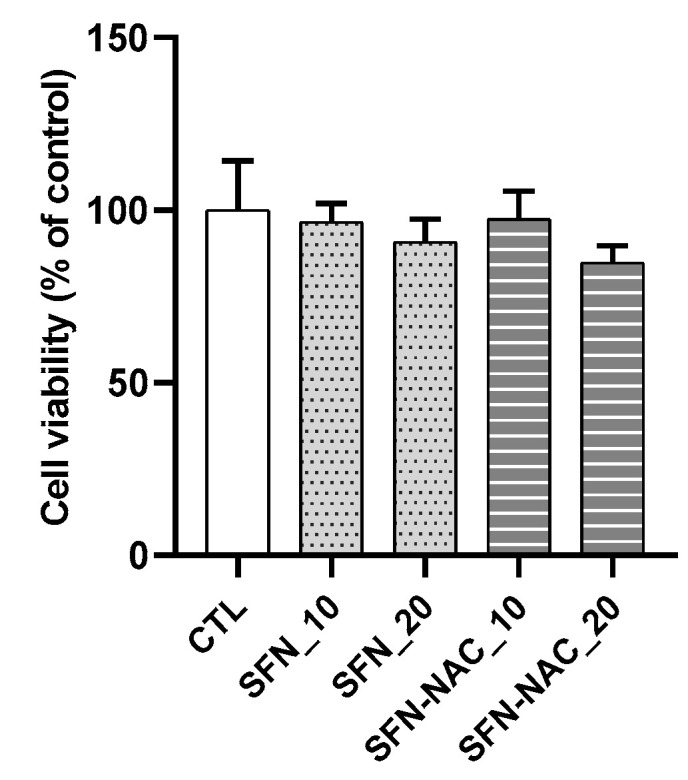
Effect of SFN and SFN-NAC on cell viability of MRC-5 fibroblast cells. The cells were treated with indicated concentrations (10 and 20 μM) of SFN and SFN-NAC for 48 h. Data values are expressed as the mean ± standard deviation of at least three different experiments. SFN, sulforaphane; SFN-NAC, sulforaphane *N*-acetylcysteine; CTL, control.

**Figure 3:**
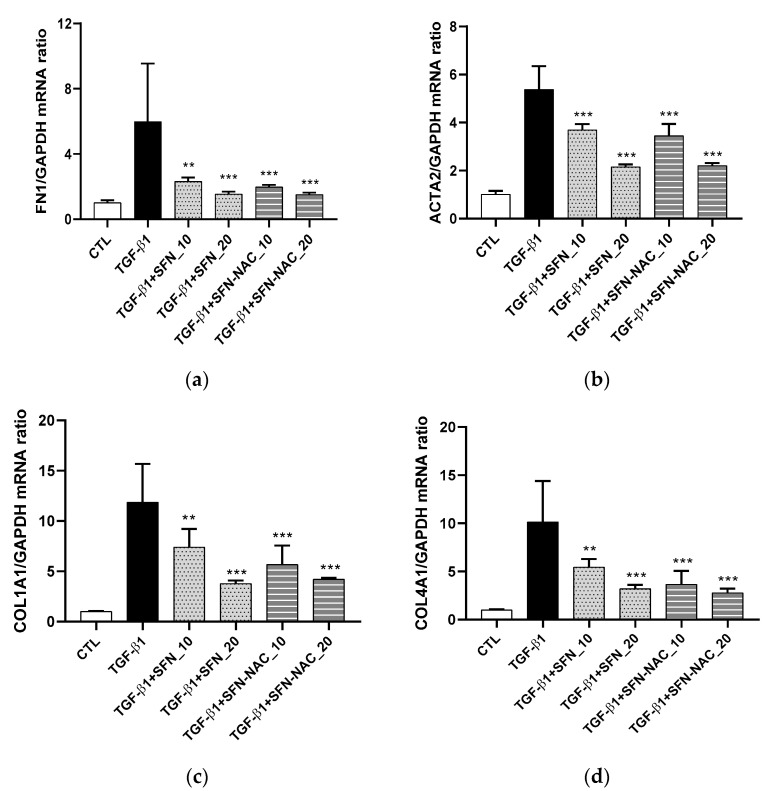
Effect of SFN and SFN-NAC treatment on the mRNA expression of ECM proteins and myofibroblast marker following TGF-β1-induced fibrosis in MRC-5 fibroblast cells. The cells were pre-treated for 1 h with the indicated concentrations of SFN and SFN-NAC, followed by treatment with TGF-β1 (5 ng/mL) for 48 h. mRNA levels of *FN1* (**a**), *ACTA2* (**b**), *COL1A1* (**c**), and *COL4A1* (**d**) were measured by qRT-PCR. Data values are expressed as the mean ± standard deviation of at least three different experiments. ** *p* < 0.01, *** *p* < 0.001 versus TGF-β1 induction. ECM, extracellular proteins; TGF-β1, transforming growth factor-β1; SFN, sulforaphane; SFN-NAC, sulforaphane *N*-acetylcysteine; CTL, control; *FN1*, fibronectin 1; *ACTA2*, actin alpha2, smooth muscle; *COL1A1*, type I collagen; *COL4A1*, type IV collagen.

**Figure 4:**
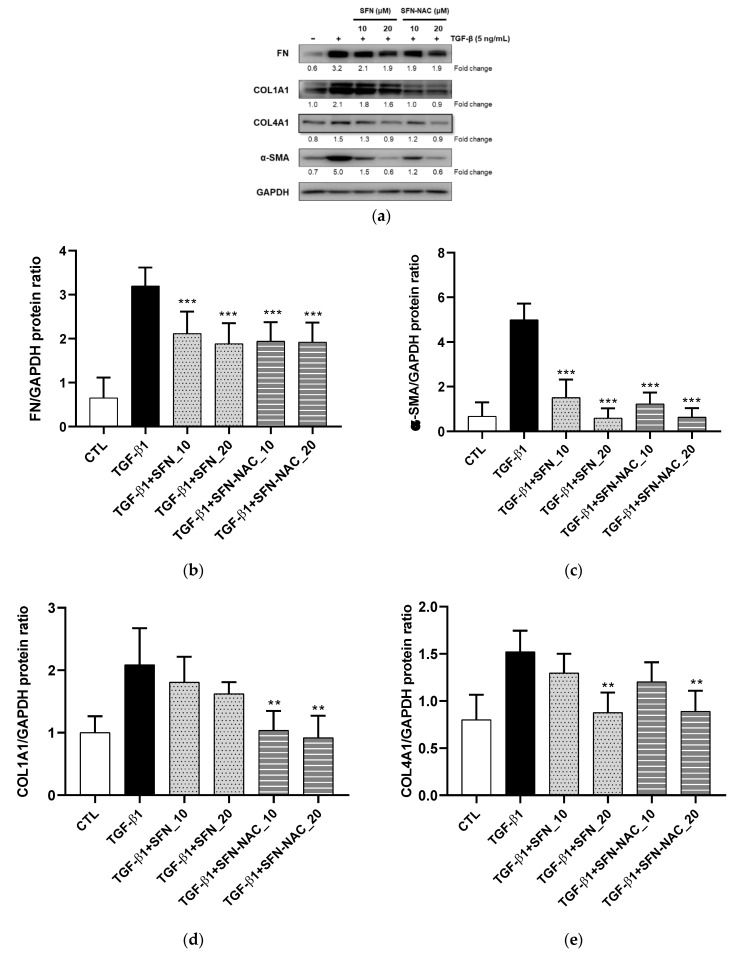
Effect of SFN and SFN-NAC treatment on the expression of ECM proteins and myofibroblast marker following TGF-β1-induced fibrosis in MRC-5 fibroblast cells. The cells were pre-treated for 1 h with the indicated concentrations of SFN and SFN-NAC, followed by treatment with TGF-β1 (5 ng/mL) for 48 h. (**a**) Western blot analysis for FN, α-SMA, COL1A1 and COL4A1. Densitometry of Western blotting of FN (**b**), α-SMA (**c**), COL1A1 (**d**), and COL4A1 (**e**). Data are expressed as the mean ± standard deviation of at least three different experiments. ** *p* < 0.01, *** *p* < 0.001 versus TGF-β1 induction. ECM, extracellular proteins; TGF-β1, transforming growth factor-β1; SFN, sulforaphane; SFN-NAC, sulforaphane *N*-acetylcysteine; CTL, control; FN, fibronectin; α-SMA, alpha-smooth muscle actin; COL1A1, type I collagen; COL4A1, type IV collagen.

We evaluated the effect of SFN and SFN-NAC on lung cell viability. Accordingly, MRC-5 cells were incubated with different concentrations of SFN and SFN-NAC (10 and 20 µM). At maximal SFN and SFN-NAC concentrations, MRC-5 cells showed no cytotoxicity (Figure 2). The deposition of ECM proteins (FN, COL1A1, and COL4A1) and myofibroblast marker (α-SMA) is considered to be a significant event in tissue remodeling and fibrosis. Therefore, the inhibitory effect of SFN and SFN-NAC on the expression levels of ECM proteins was assessed using qRT-PCR and western blot analyses. In the present study, our findings revealed that the mRNA and protein expression levels of ECM proteins, including FN, α-SMA, COL1A1, and COL1A4, were increased in TGF-β1-induced fibroblast cells. Conversely, SFN and SFN-NAC treatment significantly decreased TGF-β1-induced mRNA and protein expression levels in a dose-dependent manner (Figure 3 and Figure 4). Notably, the inhibitory effect of SFN-NAC on FN, α-SMA, and COL1A4 proteins was comparable with that mediated by SFN (Figure 4). However, SFN-NAC induced significant inhibitory effects on COL1A1 proteins; this effect was not demonstrated following the SFN treatment (Figure 4).

### 3.2. In Vitro Pharmacokinetic Properties

#### 3.2.1. Metabolic Stability of SFN and SFN-NAC

**Figure 5:**
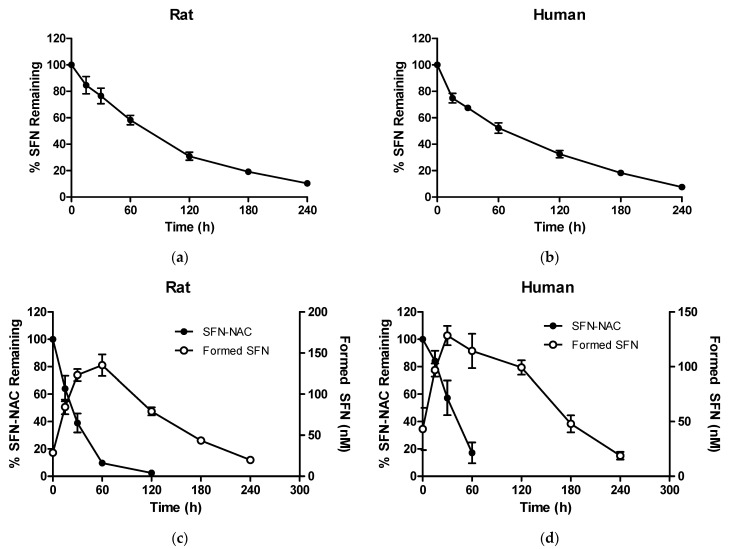
Percentages of remaining SFN and SFN-NAC as a function of time after incubation in plasma: (**a**) SFN in rat plasma; (**b**) SFN in human plasma; (**c**) SFN-NAC in rat plasma; (**d**) SFN-NAC in human plasma. Data values are expressed as the mean ± standard deviation (*n* = 3 at each point). SFN, sulforaphane; SFN-NAC, sulforaphane *N*-acetylcysteine.

SFN levels were reduced in rat and human plasma, presenting mean half-lives of 76.0 and 57.4 min, respectively (Figure 5a,b). The plasma level of SFN-NAC decreased faster than that of SFN, with mean half-lives of 23.3 and 19.0 min, respectively. In both rat and human plasma, SFN was formed after incubation with SFN-NAC. The concentration of the formed SFN increased and then decreased again in a similar manner to that following incubation with SFN (Figure 5c,d).

**Figure 6:**
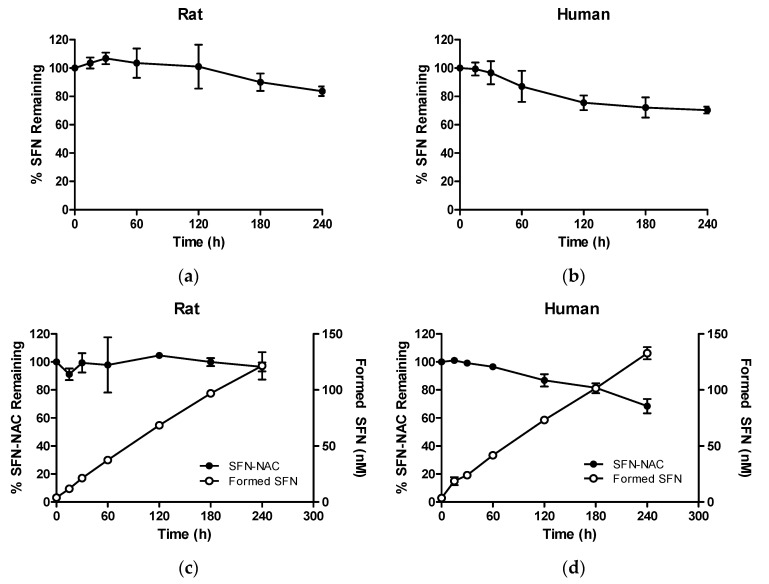
Percentages of SFN and SFN-NAC remaining as a function of time after incubation in liver microsomes: (**a**) SFN in rat liver microsomes; (**b**) SFN-NAC in rat liver microsomes; (**c**) SFN in human liver microsomes; (**d**) SFN-NAC in human liver microsomes. Data values are expressed as the mean ± standard deviation (*n* = 3 at each point). SFN, sulforaphane; SFN-NAC, sulforaphane *N*-acetylcysteine.

After liver microsomal incubation for 240 min, the decrease in SFN concentration was substantially more gradual when compared with that in plasma, with 83.7% and 70.3% remaining in rat and human microsomes, respectively (Figure 6c,d). Similarly to in plasma, SFN was formed from SFN-NAC when SFN-NAC was incubated in liver microsomes. However, unlike in plasma, the concentration of formed SFN did not decrease until 240 min, probably due to the slower metabolic rate of SFN in liver microsomes.

#### 3.2.2. Apparent Permeability for Absorption Direction in Caco-2 Cells

**Figure 7:**
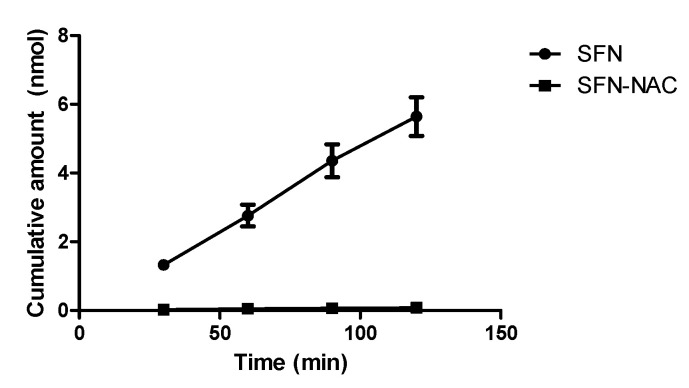
Cumulative amount of SFN and SFN-NAC transported across Caco-2 cell monolayers over time. Data values are expressed as the mean ± standard deviation (*n* = 3 at each point). SFN, sulforaphane; SFN-NAC, sulforaphane *N*-acetylcysteine.

The apical-to-basolateral transport of SFN and SFN-NAC across Caco-2 monolayers was investigated (Figure 7). Compared to that of SFN, the A-to-B transport of SFN-NAC was much lower. The calculated apparent permeability coefficients (P_app_,_A-to-B_) of SFN were 1.33 ± 0.01 × 10^−5^ cm/s, whereas the P_app_,_A-to-B_ of SFN-NAC was much lower, 0.16 ± 0.01 × 10^−6^ cm/s, suggesting a much higher intestinal absorption of SFN compared to SFN-NAC in terms of permeability.

### 3.3. In Vivo Pharmacokinetic Study at a Micro-Dose Range

#### 3.3.1. Sulforaphane

**Figure 8:**
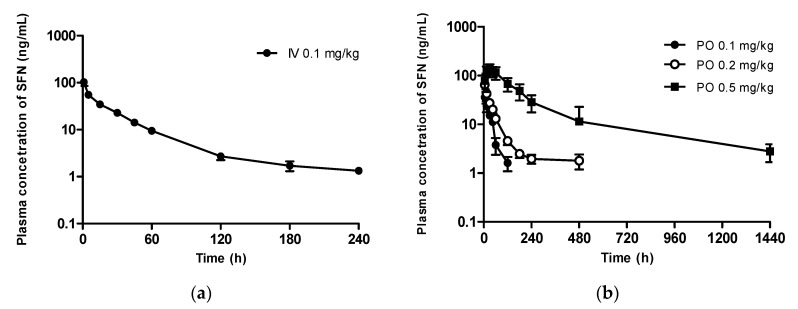
Plasma concentration of SFN after (**a**) intravenous (0.1 mg/kg) and (**b**) oral (0.1, 0.2, and 0.5 mg/kg) administration to rats. Data values are expressed as the mean ± standard deviation (*n* = 4). SFN, sulforaphane.

After intravenous administration of 0.1 mg/kg SFN, the SFN plasma concentration declined exponentially, with a terminal half-life of 61.3 min (Figure 8a and Table 1). The total body clearance of SFN was 40.7 mL/min/kg. After oral administration of SFN at 0.1, 0.2, and 0.5 mg/kg, the T_max_ ranged between 5–30 min for the three different doses. The increase in the oral AUC of SFN was more than dose-proportional, indicating non-linear pharmacokinetics of SFN even in the low dose range (Figure 8b and Table 1). Thus, the calculated oral bioavailability was similar, between 0.1 mg/kg and 0.2 mg/kg, whereas the oral bioavailability was higher than 100% at the highest dose (0.5 mg/kg).

#### 3.3.2. Sulforaphane *N*-acetylcysteine

**Figure 9:**
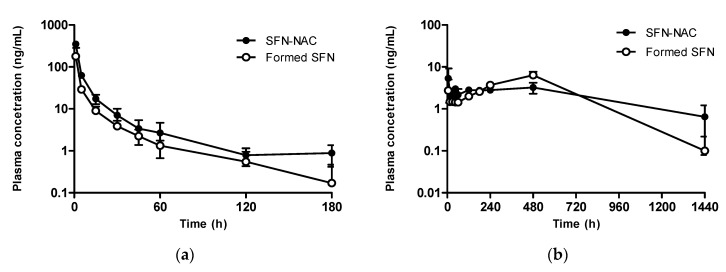
Plasma concentration of SFN-NAC and SFN after (**a**) intravenous (0.1 mg/kg) and (**b**) oral (0.5 mg/kg) administration of SFN-NAC to rats. Data values are expressed as means ± standard deviation (*n* = 4). SFN, sulforaphane; SFN-NAC, sulforaphane *N*-acetylcysteine.

After intravenous administration of 0.1 mg/kg SFN-NAC, the plasma concentration of SFN declined exponentially, presenting a terminal half-life of 33.2 min (Figure 9a and Table 2). The total body clearance of SFN-NAC was 48.3 mL/min/kg, similar to that of SFN. The plasma concentration of the formed SFN was determined after administering SFN-NAC. SFN was rapidly formed from SFN-NAC and decreased in a similar pattern and concentration range to SFN-NAC. The half-life of the formed SFN was 48.6 min, which approaches that of SFN after an oral dose of 0.1 mg/kg (Table 1 and Table 2).

After oral administration of 0.5 mg/kg SFN-NAC, the bioavailability of SFN-NAC (24.8%) was lower than that of SFN (Figure 9b and Table 2). This could be attributed to poor intestinal absorption, as the C_max_ of SFN-NAC was 0.006 μg/mL, while that of SFN was 0.143 μg/mL at identical doses. Moreover, SFN was formed after the oral administration of SFN-NAC. The concentration of SFN decreased in the early phase and then increased until 8 h after administration.

## 4. Discussion

SFN and SFN metabolites are known to possess various pharmacological activities. In the present study, our findings first revealed the antifibrotic effects of SFN and SFN-NAC, the major metabolite of SFN. Furthermore, the pharmacokinetic profiles of SFN and SFN-NAC were evaluated to confirm their potential as therapeutic agents for pulmonary fibrosis.

In the present study, we observed that SFN and SFN-NAC inhibited TGF-β1-induced fibrosis in pulmonary fibroblasts. Both SFN and SFN-NAC significantly inhibited the overexpression of fibrosis-related proteins, including FN, α-SMA, COL1A1, and COL4A1, induced by TGF-β1 in fibroblast cells without observed cytotoxicity (Figure 3 and Figure 4). TGF-β, a potent fibrotic mediator, acts on multiple cell types during fibrosis, inducing fibroblast proliferation and differentiation into myofibroblasts [25,26,27]. In addition, the mRNA expression of these proteins was decreased following SFN and SFN-NAC treatment. FN, α-SMA, and collagens are major fibrous proteins that compose the ECM and play crucial roles in cell adhesion, including differentiation, growth, migration, and wound healing [28]. Excessive deposition contributes to the pathogenesis of pulmonary fibrosis [25,28]. Both SFN and SFN-NAC presented similar inhibitory effects mediated by anti-pulmonary fibrosis-related genes (Figure 3 and Figure 4), suggesting that the antifibrotic effect could be sustained even after SFN metabolism to SFN-NAC, the final major metabolite, by administering SFN-containing food or SFN itself.

Therefore, to accurately understand the pharmacological effects of SFN and SFN-NAC, we evaluated the pharmacokinetics of both SFN and SFN-NAC. Both SFN and SFN-NAC were stable during liver metabolism, but their concentration rapidly decreased in the plasma, indicating that the rapid clearance of SFN might be due to plasma metabolism. The volume of distribution of SFN was moderate, implying that SFN could be distributed to target tissues such as the lung and liver to exhibit antifibrotic effects. Following oral administration, the bioavailability was over 70%; sufficient for development as a therapeutic agent. As large variabilities in pharmacokinetic parameters of SFN have been reported in previous studies, we evaluated the pharmacokinetics of SFN at low doses, ranging from 0.1 to 0.5 mg/kg. The pharmacokinetic parameters of SFN presented linear kinetics up to 0.2 mg/kg; however, at 0.5 mg/kg, the drug exposure exceeded the proportional dose (Table 1), which could be attributed to the saturable metabolism of SFN and/or reconversion of SFN-NAC to SFN. Notably, in the high dose range assessed in a previous pharmacokinetic study, the dose-normalized AUC and SFN bioavailability decreased in the range of 0.5 to 5 mg/kg, possibly due to saturation of the absorption process [21]. After intravenous and oral administration of SFN-NAC, rapid formation of SFN was confirmed with a similar decrease in SFN, indicating rapid equilibrium in the reversible metabolism between SFN and SFN-NAC.

Despite its various therapeutic benefits, SFN presents several problems that need to be resolved prior to oral administration. Broccoli sprout extracts containing glucosinolates (the precursor of SFN) and SFN have shown good tolerability and no significant side effects in humans [29]. However, gastrointestinal side effects such as bloating, diarrhea, dyspepsia, and flatulence have been observed on administering a large SFN dose [30]. Furthermore, Lozanovski et al. have reported that broccoli sprouts can cause digestive issues, such as vomiting and nausea [31]. In terms of pharmacokinetics, as SFN itself is unstable, it is difficult to maintain an efficacious concentration for a sufficient period. Based on the finding that SFN-NAC is converted to SFN, an SFN-NAC prodrug could be an alternative approach to overcome the above challenges, although SFN-NAC is also a major metabolite of SFN. Notably, it was first demonstrated that SFN could be formed from SFN-NAC in plasma and liver microsomal stability studies, possibly due to the deconjugation reaction (Figure 5 and Figure 6). Moreover, upon SFN-NAC administration via intravenous or oral administration, the formed SFN was rapidly converted and maintained in vivo along with SFN-NAC. However, the absolute oral bioavailability of SFN-NAC was found to be low (>24.8%) when compared with that following SFN administration (Table 1 and Table 2), indicating that chemical modification is crucial for enhancing intestinal absorption when developing SFN-NAC as a novel drug candidate. Moreover, considering that SFN-NAC, the major SFN metabolite, undergoes gastrointestinal absorption after conversion to SFN-NAC, the reversible metabolism between SFN-NAC and SFN could lead to long-term pharmacological effects, as afforded by antifibrotic agents in the lungs.

## 5. Conclusions

The anti-pulmonary fibrotic effects, as well as the in vitro and in vivo pharmacokinetics of SFN and SFN-NAC, a major metabolite of SFN, were evaluated to elucidate the effects of SFN-containing products. Our findings revealed that SFN and SFN-NAC present similar antifibrotic activities. Furthermore, as SFN-NAC is reconverted to SFN in the body, SFN-NAC can be potentially developed as an alternative to overcome the gastrointestinal side effects associated with SFN. If the low absorption of SFN-NAC can be improved by chemical modification in the future, SFN-NAC could be a promising therapeutic agent to achieve a prolonged duration of action, with reduced side effects of SFN and its metabolites.

## Figures and Tables

**Table 1:** Pharmacokinetic parameters of SFN after intravenous (0.1 mg/kg) and oral (0.1, 0.2, and 0.5 mg/kg) administration to rats. Data values are expressed as the mean ± standard deviation (*n* = 4).

Parameters	IV		PO	
	0.1 mg/kg	0.1 mg/kg	0.2 mg/kg	0.5 mg/kg
AUC_last_ (μg·min/mL)	2.35 ± 0.154	1.85 ± 0.377	3.16 ± 0.073	29.8 ± 8.64
AUC_inf_ (μg·min/mL)	2.47 ± 0.205	1.92 ± 0.411	3.57 ± 0.253	31.0 ± 8.89
t_1/2_ (min)	61.3 ± 36.2	41.9 ± 7.60	197.7 ± 169	320 ± 75.4
MRT (min)	56.2 ± 11.7			
C_max_ (μg/mL)	-	0.036 ± 0.010	0.065 ± 0.012	0.143 ± 0.035
T_max_ (min)	-	15.0 ± 0.0	5.0 ± 0.0	30.0 ± 18.4
V_ss_ (mL/kg)	2268 ± 337			
CL (mL/min/kg)	40.7 ± 3.4			
F (%)		77.8%	72.4%	251.4%

SFN, sulforaphane; IV, intravenous; PO, peroral; AUC_last_, area under the plasma concentration-time curve from time zero to the last measured time; AUC_inf_ area under the plasma concentration-time curve from time zero to infinity; t_1/2_, elimination half-life; MRT, mean residence time; C_max_, maximum serum drug concentration; T_max_ time take to reach C_max_; V_ss_, volume of distribution at steady-state; CL, total body clearance; F(%), oral bioavailability, i.e., the fraction of an orally administered drug that reaches systemic circulation.

**Table 2:** Pharmacokinetic parameters of SFN-NAC and SFN after intravenous (0.1 mg/kg) and oral (0.5 mg/kg) administration of SFN-NAC to rats. The data are expressed as the mean ± standard deviation (*n* = 4).

Parameters	IV (SFN-NAC 0.1 mg/kg)		PO (SFN-NAC 0.5 mg/kg)	
	SFN-NAC	SFN	SFN-NAC	SFN
AUC_last_ (μg·min/mL)	2.11 ± 0.502	1.07 ± 0.215	2.67 ± 1.22	1.73 ± 0.186
AUC_inf_ (μg·min/mL)	2.16 ± 0.515	1.11 ± 0.233	NC	NC
t_1/2_ (min)	33.2 ± 14.2	48.6 ± 11.0	NC	NC
MRT (min)	15.8 ± 7.7			
C_max_ (μg/mL)	-		0.006 ± 0.003	0.006 ± 0.001
T_max_ (min)	-			
V_ss_ (mL/kg)	711 ± 262	-	-	-
CL (mL/min/kg)	48.3 ± 10.5	-	-	-
F(%)	-	-	>24.8%	-

SFN, sulforaphane; SFN-NAC, sulforaphane *N*-acetylcysteine; IV, intravenous; PO, peroral; AUC_last_, area under the plasma concentration-time curve from time zero to the last measured time; AUC_inf_ area under the plasma concentration-time curve from time zero to infinity; t_1/2_, elimination half-life; MRT, mean residence time; C_max_, maximum serum drug concentration; T_max_ time taken to reach C_max_; V_ss_, volume of distribution at steady-state; CL, total body clearance; F(%), oral bioavailability, i.e., the fraction of an orally administered drug that reaches systemic circulation.

## Data Availability

The data presented in this study are available in the article.
